# Effects of Resveratrol Administration in Liver Injury Prevention as Induced by an Obesogenic Diet: Role of Ruminococcaceae

**DOI:** 10.3390/biomedicines10081797

**Published:** 2022-07-26

**Authors:** Iñaki Milton-Laskibar, Amanda Cuevas-Sierra, María P. Portillo, J. Alfredo Martínez

**Affiliations:** 1 Precision Nutrition and Cardiometabolic Health, IMDEA-Food Institute (Madrid Institute for Advanced Studies), Campus of International Excellence (CEI) UAM+CSIC, Spanish National Research Council, 28049 Madrid, Spain; inaki.milton@imdea.org (I.M.-L.); amanda.cuevas@imdea.org (A.C.-S.); jalfredo.martinez@imdea.org (J.A.M.); 2 CIBER Fisiopatología de la Obesidad y Nutrición (CIBEROBN), Instituto de Salud Carlos III (ISCIII), 28222 Madrid, Spain; 3 BIOARABA Institute of Health, 01006 Vitoria-Gasteiz, Spain; 4 Nutrition and Obesity Group, Department of Pharmacy and Food Sciences, Faculty of Pharmacy and LuciLascaray Research Center, University of the Basque Country (UPV/EHU), 01006 Vitoria-Gasteiz, Spain

**Keywords:** resveratrol, microbiota, NAFLD, fructose, *Ruminococcaceae*

## Abstract

Gut microbiota dysbiosis has been described in several metabolic disruptions, such as non-alcoholic fatty liver disease (NAFLD). Administration of resveratrol has been claimed to elicit benefits against NAFLD along with modulating gut microbiota composition. This investigation aims to study the putative mediating role of gut microbiota in the potential hepato-protective effects of resveratrol in a diet-induced NAFLD rat model. The involvement of bacteria from the *Ruminococcaceae* family in such effects was also addressed. Resveratrol administration resulted in lowered liver weight and serum total and non-HDL cholesterol concentrations, as well as in increased serum HDL cholesterol levels. The administration of this polyphenol also prevented obesogenic diet-induced serum transaminase increases. In addition, histopathological analysis revealed that resveratrol administration ameliorated the dietary-induced liver steatosis and hepatic inflammation. Gut microbiota sequencing showed an inverse relationship between some bacteria from the *Ruminococcaceae* family and the screened hepatic markers, whereas in other cases the opposite relationship was also found. Interestingly, an interaction was found between *UBA-1819* abundance and resveratrol induced liver weight decrease, suggesting that for this marker resveratrol induced effects were greater when the abundance of this bacteria was high, while no actions were found when *UBA-1819* abundance was low.

## 1. Introduction

Gut microbiota is a complex community of microorganisms residing in the gastrointestinal tract that can reach concentrations of almost a trillion cells per gram and may weight up to 2 kg in a normal weight human being [1,2]. Despite virus and fungi occurring in the intestine, gut microbiota is mainly composed of bacteria, being *Bacteroidetes*, *Clostridium*, *Prevotella*, *Eubacterium*, *Ruminococcus*, *Fusobacterium*, *Peptococcus*, and *Bifidobacterium* as the most abundant genera [3]. Of note, gut microbiota exerts an individualized influence on several physiological processes in the host, playing a major role in the processing of non-digestive polysaccharides, the synthesis of certain vitamins and metabolites (postbiotics) and the regulation of energy-balance and cell metabolism, as well as in immunocompetence and gut barrier maintenance [4,5]. The involvement of gut microbiota in these processes may be challenged in situations where microorganisms’ distribution and composition are impaired. Indeed, gut microbiota abundance and diversity are dynamic and can be affected by factors such as the hosts’ genetic make-up and age or the administration of antibiotics [6]. In addition, nutrition has emerged as a main modulator of gut microbiota composition. Diets rich in vegetables, fruits, and legumes, and thus providing high proportions of fibre, are considered to boost bacterial diversity [5]. Contrarywise, dietary patterns characterized by high contents of fats and/or refined carbohydrates and providing low fibre amounts (as occurs in “westernized” diets) can lead to an impaired gut microbiota diversity and abundance [7]. Indeed, gut microbiota dysbiosis also occurs in several chronic metabolic diseases such as obesity or type 2 diabetes, showing that it is a factor involved in their onset and/or development [8].

In this context, non-alcoholic fatty liver disease (NAFLD) is a condition where gut microbiota impairment can be a potential mediating mechanism [9]. Currently, NAFLD is considered the most prevalent liver pathological condition in western societies, being also the main contributor for the development of further chronic hepatic alterations [10]. In fact, insulin resistance plays a major role in the development of this liver alteration, triggering a myriad of processes, such as increased hepatic *de novo* lipogenesis, liver mitochondrial dysfunction or disturbed white adipose tissue lipolysis [9]. Among the causes leading to NAFLD, excessive fructose intake stands as one of the main mediators, with a recognized ability to promote lipid deposition in the liver and to enhance *de novo* lipogenesis [11].

Regarding the role of gut microbiota on NAFLD, dysbiosis has been reported to result in a greater intestinal permeability, as well as an enhanced release of pro-inflammatory mediators to the bloodstream. These pro-inflammatory molecules may reach the liver, activate inflammatory pathways, and enhance the release of inflammatory cytokines [12]. It is noteworthy that excessive fructose consumption has been reported to affect gut microbiota composition and alter intestinal permeability in rodents [13]. In this line, decreased microbial diversity and increased abundance of ethanol-producing bacteria have been described in NAFLD [14]. These fructose-induced gut microbiota impairments also affect metabolite production, such as short-chain fatty acids (SCFA), which in turn may alter hepatic lipid metabolism [15].

In this scenario, the administration or intake of natural bioactive compounds has long been proposed as a complementary/alternative therapeutic tool for treating metabolic diseases including NAFLD treatment. Indeed, effective conventional interventions (based in hypocaloric diets) for NAFLD management [16], showed a limitation, such as the low adherence of subjects, especially in the long term. Moreover, due to the link between microbiota dysbiosis and NAFLD, therapies targeting gut microbiota have been proposed as potential interventions for the management and prevention of this liver condition [17]. Resveratrol (3,5,4′-trihydroxy-*trans*-stilbene) is a widely studied bioactive compound with proven effectiveness in NAFLD management [18,19,20,21] and with the capacity to modulate gut microbiota composition [5]. The low bioavailability of resveratrol needs to be reconsidered [22], since once ingested, resveratrol undergoes extensive metabolization, by phase II enzymes in the gut and the liver, and by the gut microbiota, and thus, the amount of intact polyphenol reaching the liver is limited [22]. Noteworthy, both the intact resveratrol that reaches the gut, and the metabolites generated, are considered to exert a “prebiotic-like” effect, modulating gut microbiota composition [23]. Indeed, it cannot be ruled out that part of the hepato-protective effects attributed to resveratrol (and other polyphenols) may well be facilitated by specific effects on gut microbiota or in the synthesis of postbiotics [24,25].

The aim of the current investigation is to study the putative mediating role of gut microbiota in the potential hepato-protective effects of resveratrol in a diet-induced rat model of NAFLD, paying special attention to the eventual involvement of bacteria from the *Ruminococcaceae* family in such metabolic effects.

## 2. Materials and Methods

### 2.1. Animals, Diets and Experimental Design

A total of 30 male six-week-old male Wistar Wistar RccHan rats (Envigo, Barcelona, Spain) were used in this study. All the experimental procedures were performed according to the guidelines of the Ethical Committee of the University of the Basque Country (document reference CUEID CEBA/30/2010), in agreement with the European regulations (European Convention-Strasburg 1986, Directive 2003/65/EC and Recommendation 2007/526/EC).

Animals were housed in polycarbonate metabolic cages (Tecniplast Gazzada, Buguggiate, Italy) in a room with controlled temperature (22 °C) and a 12 h light/dark cycle. After a 6-day adaptation period, rats were randomly allocated into 3 experimental groups (10 animals each). The control group (C group) was fed a standard diet (AIN-93G, OpenSource Diets, Denmark, D10012G) and the remaining animals were fed a high-fat high-fructose diet (OpenSource Diets, Denmark, D09100301) alone (HFHF group) or supplemented with resveratrol at a dose of 30 mg/kg bw/day (RSV group). Resveratrol was kindly supplied by Monteloeder (Elche, Alicante, Spain), and daily incorporated into the powdered diets as previously reported [26]. The rodents were maintained under these experimental conditions for eight weeks with free access to food and water. Resveratrol was daily added to the diet (diluted in ethanol), whereas the controls (C and HFHF) received the vehicle. At the end of the whole experimental period, animals were sacrificed (cardiac exsanguination) under anaesthesia (chloral hydrate) (Sigma-Aldrich, Saint Louis, MO, USA).

Body weight and food intake were daily monitored. Serum was obtained by centrifugation of blood samples after clotting (1000× *g* for 10 min, at 4 °C). Livers were dissected, weighed, and immediately frozen in liquid nitrogen. All samples were stored at −80 °C until analyses.

### 2.2. Histopathological Evaluation of NAFLD

After sacrifice, liver samples were fixed in 10% buffered formalin and subsequently embedded in paraffin for histological study by light microscopy as previously reported [27]. Haematoxylin and eosin along with Masson’s trichrome were used to stain liver sections using standard techniques. During the analysis of liver sections, hepatologists were blinded regarding the group that each animal/sample belonged to.

Lobular inflammation was graded according to the number of inflammatory foci per 200× field. Thus, a value of 0 was assigned when inflammatory foci did not appear, a value of 1 when there were 1 to 2 inflammatory foci, and 2 when 2 to 4 inflammatory foci were found. Ballooning damage was evaluated based on the number of hepatocytes featuring this type of injury. In this case, a value of 0 was given when there was no ballooning, a value of 1 when ballooning was observed in few hepatocytes, and a value of 2 when many hepatocytes exhibited ballooning degeneration. Finally, NAS score was calculated by means of data from steatosis, lobular inflammation, ballooning, and fibrosis scores.

### 2.3. Serum Biochemistry

Blood samples were centrifugated (1000× *g* for 10 min, at 4 °C) and serum component was used for biochemical determinations. Commercial spectrophotometric kits were used to measure serum alanine aminotransferase (ALT) and aspartate aminotransferase (AST) levels, following the supplier guidelines (Biosystems, Barcelona, Spain). Total and HDL-cholesterol serum levels were also analysed by spectrophotometry based commercial kits (Biosystems, Barcelona, Spain), whereas non-HDL cholesterol levels were estimated by subtracting HDL cholesterol levels to those of total cholesterol, as described elsewhere [28].

### 2.4. Faecal DNA Extraction and 16S rRNA Gene Amplification

Faecal samples were collected in a Falcon tube the day before sacrifice (prior overnight fasting) housing the animals separately and inducing defecation with an abdominal massage. Samples were stored at −80 °C. The QIAamp DNA stool MiniKit was used to extract DNA from faecal samples following the manufacturer’s instructions (QIAGEN, Hilden, Germany). Microbiota composition was assessed by amplifying the variable V3 and V4 regions of the bacterial 16S ribosomal RNA gene (16S rRNA) from the faecal DNA and sequenced with the Illumina MiSeq platform (Illumina, San Diego, CA, USA) as explained elsewhere [27].

The Quantitative Insights Into Microbial Ecology program (QIIME2) [29] was used to process the 16S rRNA gene sequence data. Low-quality reads were filtered, and subsequently chimeric sequences were removed. DADA2 [30] was used to cluster clean reads as amplicon sequence variants (ASVs), which were annotated with the SILVA v.132 16S rRNA gene reference database [31]. Relative abundance of each ASV was calculated using the Phyloseq R package. Microbiome Analyst platform [32] was used for alpha diversity calculation through the Shannon index and comparisons between groups were performed by Kruskal—Wallis tests. Beta diversity was calculated using Bray—Curtis index and PERMANOVA test, depicted in a non-multidimensional dimension scale (NMDS). Data were normalized by the centered log-ratio (CLR) method [33].

### 2.5. Statistycal Analysis

Statistical analyses were performed using SPSS 24.0 (SPSS, Chicago, IL, USA). Descriptive results are presented as mean ± SEM. Data were analysed by one-way ANOVA, followed by Newman—Keuls post-hoc test. In the case of differences in alpha diversity, comparisons between groups were performed by Kruskal–Wallis test. Beta diversity was calculated using Bray—Curtis index and PERMANOVA test, depicted in a non-multidimensional dimension scale (NMDS). Significance was assessed at the *p* < 0.05 level.

## 3. Results

### 3.1. Somatometric Variables, Serum Biochemistry and Liver Histological Analysis

**Figure 1:**
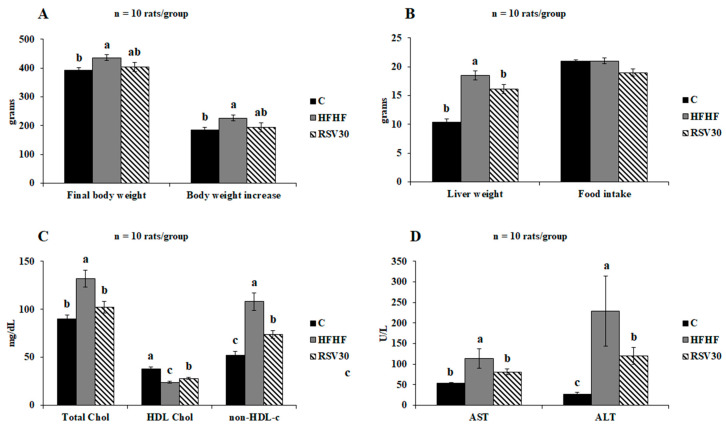
Final body weight and body weight increase (**A**), liver weight and food intake (**B**), serum total, HDL, and non-HDL cholesterol levels (**C**) and serum aminotransferase levels (**D**) in rats fed a control diet (**C**), a high-fat high-fructose diet (HFHF) or a high-fat-high-fructose diet supplemented with 30 mg/kg/d resveratrol (RSV30). Values are presented as mean ± SEM. Bars not sharing common letters are significantly different (*p* < 0.05). ALT: alanine aminotransferase, AST: aspartate aminotransferase.

The high-fat high-fructose diet feeding resulted in a statistically significant body weight increase (*p* < 0.01), as well as a significantly greater final body weight (*p* < 0.01), compared to the control group (Figure 1A). In those animals fed the obesogenic diet and treated with resveratrol, a slight decrease was found in both the body weight gain and the final body weight of the animals, though without reaching statistical significance. By contrast, the increased liver weight found in the animals fed the high-fat high-fructose diet alone was effectively prevented by resveratrol administration (*p* < 0.05). Noteworthy, all these effects were found without apparent differences in food intake (Figure 1B).

Concerning the analysed serum markers, the statistically significant increase in total cholesterol levels induced by the high-fat high-fructose diet (*p* = 0.001 vs. control group) was effectively reverted by resveratrol administration, reaching values comparable to those observed in the control group. Similarly, the reduction induced by the obesogenic diet in circulating HDL-cholesterol levels was significantly higher in the animals supplemented with resveratrol, although in this case the observed levels were still significantly higher than those found in the control animals (Figure 1C). Finally, the subtraction of HDL cholesterol levels to those of total cholesterol revealed that this biochemical variable, which was significantly increased in the HFHF group compared to the control group, was lowered by resveratrol supplementation, although without reaching the control group value (Figure 1C). Serum transaminase (AST, ALT) levels were significantly increased in the animals fed the high-fat high-fructose diet alone compared to the control group. In both cases, resveratrol supplementation led to significantly lower values of these variables in comparison to the ones found in the HFHF group. Indeed, in the case of AST levels, resveratrol-mediated reduction resulted in values similar to those found in the control animals (Figure 1D).

As reported elsewhere, liver histology analyses revealed that all the animals in the control group had no steatosis at all (Table 1) [27]. By contrast, the high-fat high-fructose feeding resulted effective in inducing steatosis. In this regard, most of the animals fed the obesogenic diet alone featured grade 2 or 3 steatosis. Interestingly, the animals fed this same diet and were supplemented with resveratrol showed a significant improvement compared to the animals in the HFHF group. In this case, most animals developed a grade 1 steatosis, demonstrating the protective effect exerted by resveratrol administration (Table 1). Related to liver inflammation, the animals in the control group, and thus fed the standard diet, showed no liver inflammation. Moreover, in these circumstances the high-fat high-fructose diet resulted in a mild-to-moderate liver inflammation. As far as resveratrol-supplemented animals are concerned, the effectiveness of the polyphenol resulted more limited than in the case of steatosis prevention, since the inflammatory injury found in the livers of these animals were similar to those observed in the HFHF group (Table 1).

### 3.2. Dietary Induced Shifts in Microbiota Composition

**Figure 2:**
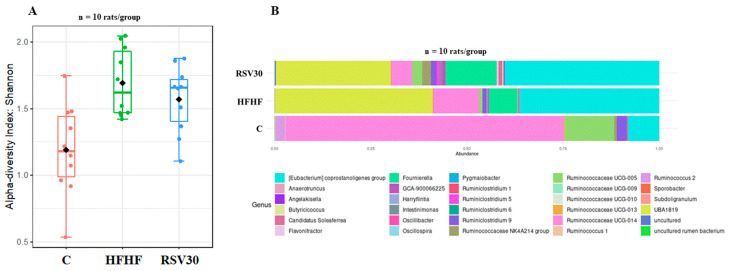
Microbial diversity according to the Shannon index (**A**) and histogram representing the relative abundances of bacteria within the *Ruminococcaceae* family (**B**) measured in faecal samples in rats fed a control diet (**C**), a high-fat high-fructose diet (HFHF) or a high-fat-high-fructose diet supplemented with 30 mg/kg/d resveratrol (RSV30).

To analyse the potential changes induced by the high-fat high-fructose diet and resveratrol administration in gut microbiota composition, the alpha diversity was assessed by the Shannon index (Figure 2A). Remarkable differences were found between control and the HFHF and RSV groups, showing that alpha diversity in control rats was significantly lower (*p* = 0.006). By contrast, no differences were found in this variable between the HFHF and the RSV30 group. In addition, the animals fed on this diet were also characterized by a greater relative abundance of *Eubacterium corpostalonigenes* specie, *Fourneriella* and *UBA-1819*. As far as resveratrol administration is concerned, the microbial composition of rats receiving the polyphenol was comparable to that observed in the non-supplemented high-fat high-fructose diet-fed animals (Figure 2B).

**Figure 3:**
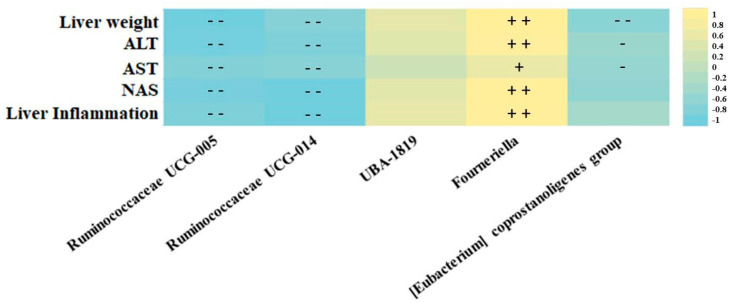
Representative heatmap of the Spearman correlation values of the most abundant *Ruminococcaceae* family microbes and studied liver damage markers. +/− = *p* < 0.05, + +/− − = *p* < 0.01.

After screening the effects of the high-fat high -fructose diet, supplemented or not with resveratrol on gut microbiota composition, correlation analyses evidenced an association between the markers of liver damage and the function, and the most abundant microbes from the *Ruminococcaceae* family (Figure 3). In this regard, the *Ruminococcaceae* UCG-014 and UCG-005 genera were associated with a hepatoprotective effect, since a strong negative correlation was found between these bacteria and variables such as liver weight, serum transaminase levels (AST and ALT) and liver histopathological markers (steatosis grade and liver inflammation). Moreover, *Fourneriella* and *UBA-1819* were positively associated with liver damage since strong direct correlations were found between the relative abundances of these microbes and the aforementioned markers of liver damage and function (Figure 3). Interestingly, *UBA-1819* relationships with liver markers did not show any statistical association.

### 3.3. Association of Ruminococcaceae Abundance with Markers of Liver Damage

**Figure 4:**
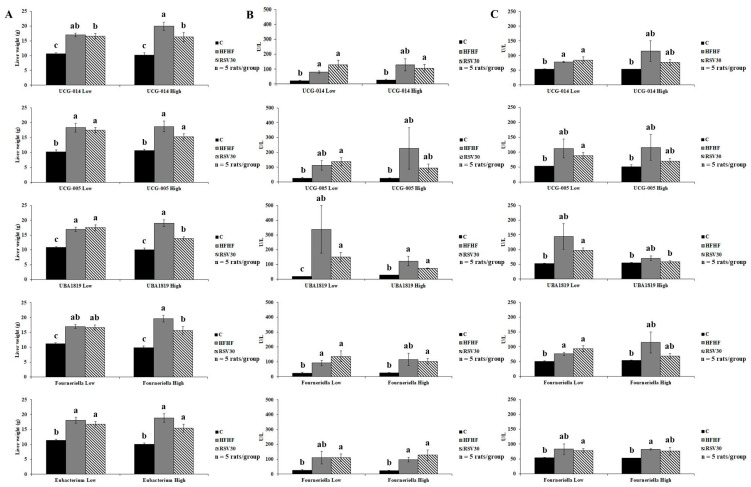
Graphic representation of liver weight (**A**), serum ALT (**B**) and serum AST (**C**) levels according to the relative abundance of *Ruminococcaceae* UCG-014, *Ruminococcaceae* UCG-005, *UBA-1819*, *Fourneriella* and *Eubacterium corpostalonigenes* in rats fed a control diet (**C**), a high-fat high-fructose diet (HFHF) or a high-fat-high-fructose diet supplemented with 30 mg/kg/d resveratrol (RSV30). Values are presented as mean ± SEM. Bars not sharing common letters are significantly different (*p* < 0.05). ALT: alanine aminotransferase, AST: aspartate aminotransferase.

To better elucidate the relationship between the relative abundance of specific bacteria from the *Ruminococcaceae* family and markers of liver status, potential interactions were analysed (Figure 4). First the animals within each of the three experimental groups were stratified, according to their abundances in *Ruminococcaceae* UCG-014, UCG-005, *UBA-1819*, *Fourneriella* and *Eubacterium corpostalonigenes*, as high or low (above or below the median for each of the studied bacteria, respectively). Then, potential interactions between *Ruminococcaceae* abundance and liver weight and serum transaminase levels were statistically tested (Figure 4A–C). According to this approach, a significant interaction was found for *UBA-1819* abundance and liver weight (*p* = 0.006). In this regard, a higher *UBA-1819* abundance was associated with a lower liver weight in animals fed the high-fat high-fructose diet and treated with resveratrol (Figure 4A). Thus, for this variable, it could be suggested that the response to resveratrol administration of the animals featuring a higher abundance of this bacteria is greater than that of animals with lower relative abundance.

## 4. Discussion

The potential involvement of the gut microbiota composition in the onset and development of an array of metabolic disturbances (including obesity, dyslipidemia, or hyperglycaemia) is a research topic that has attracted a great deal of interest in the last few years [34,35,36]. Similarly, the role of gut microbiota signatures in the response of a subject to specifically prescribed therapeutic diets or food bioactives has also become relevant in the research field of personalized nutrition [37]. Therefore, understanding the way that shifts in gut microbiota composition (induced by diet or potential therapeutic food components) are related to different metabolic outcomes is a major scientific endeavour.

According to current investigations, a greater abundance of ethanol-producing bacterial species is common in individuals featuring NAFLD, resulting in increased circulating ethanol concentrations, which in turn may trigger pro-inflammatory pathways in the liver [38]. Additionally, the microbiota of subjects showing NAFLD is typically characterized by a higher abundance of *Proteobacteria* at the phylum level, higher abundance of *Enterobacteriaceae* and decreased abundance of *Rikenellaceae* and *Ruminococcaceae* at the family level, as well as increased abundance of *Escherichia*, *Dorea* and *Peptoniphilus*, and decreased abundance of *Anaerosporobacter*, *Coprococcus*, *Eubacterium*, *Faecalibacterium* and *Prevotella* at the genus level [39,40]. Insulin resistance, which plays a key role in NAFLD aetiology [41], is characterized by a loss of butyrate-producing bacteria and a lowered relative abundance of *Akkermansia muciniphila*, as well as enhanced abundance of bacteria with pro-inflammatory potential [42,43]. In this line, rats fed the high-fat high-fructose diet included in this experiment trial showed a decreased gut microbiota diversity compared to control animals, as reported elsewhere [27].

Some of the health benefits described for resveratrol administration may be attributable, at least in part, to specific effects in gut microbiota composition [44]. Thus, it has been reported that intestinal microbiota was involved in the favourable effects of resveratrol in a diet-induced NAFLD mouse model, where resveratrol not only reduced liver fat accumulation and insulin resistance, but also significantly changed gut microbiota richness, diversity and variability, mediating the adaptation of metabolic pathways related to lipid and glucose turnover [45]. Moreover, in such trials, correlation analyses suggested that the improvements in NAFLD metabolic indicators were narrowly related to the altered gut microbiota, where resveratrol may putatively exert some hepatoprotective effects [45]. Other research, focused on the beneficial influence of resveratrol on the amelioration of NAFLD in mice, found that besides decreased body weight, systemic low-grade inflammation and liver steatosis, the administration of this polyphenol modulated gut microbiota composition by decreasing the abundance of bacteria, such as *Desulfovibrio*, *Lachnospiraceae*_*NK4A316*_group and *Alistipes*, and increased the abundance in SCFA producing bacteria, such as *Allobaculum*, *Bacteroides* and *Blautia* [46]. Other trials performed in high-fat diet-fed rodents, evidenced that the health effects of resveratrol administration on intestinal tight junction proteins were accompanied not only by some benefits on lipid and glucose profiles, inflammation, endotoxemia and intestinal integrity disruptions, but also by a significant reduction in bacteria from the *Ruminococcus* genera [47]. In our case, resveratrol administration did not result in significant changes in gut microbiota diversity [27]. This apparent lack of effect may be related to the animal model and the experimental conditions (selected resveratrol dose and diet), as proposed by other authors [5].

Considering that this is a complementary study to a previously published investigation [27] where a greater abundance of genera within the *Ruminococcaceae* family was found in the control group, the present study was devoted to gaining more insight into the relationship between changes in the bacteria family and the prevention of liver steatosis induced by resveratrol. Indeed, the greater abundances of *Ruminococcaceae* UCG-014 and UCG-005 observed in the faeces of the animals in the control group were significantly reduced by the high-fat high-fructose feeding pattern. In this line, the relationship of these bacteria with several metabolic disturbances, including NAFLD, has been highlighted in previous studies [48,49,50]. In the present study, a negative association was found between several markers of liver status, including liver weight, serum transaminase levels, steatosis grade or hepatic inflammation, and the abundance of certain members of the *Ruminococcaceae* family, such as *Ruminococcaceae* UCG-005 or *Ruminococcaceae* UCG-014; whereas *Fourneriella* showed to be positively associated with liver damage. Interestingly, an interactive pattern was found when analysing the association between the aforementioned markers of liver status and the relative abundance of *UBA-1819*. In this case, the abundance of this bacteria seems to somehow modulate the response to resveratrol administration. According to our results, the animals featuring a higher relative abundance of *UBA-1819* showed a greater response to the polyphenol administration than those featuring lower levels, as revealed by the lower organ weight found in these animals. This pattern could have been more evident with a higher number of animals in the experimental groups. In any case, the outcomes for *UBA-1819* should be considered with attention since faecal levels of this microorganism may have a dual role on liver status, depending on gut abundance and therefore a potential interactive influence should be considered in the future.

The results achieved in the present study agree with those previously reported by other authors. Thus, a study exploring adipose tissue inflammation and gut microbiota interactions revealed that the administration of Polydextrose to obese mice (fed a high-fat diet) promoted the growth of microbes including *Ruminococcaceae* UCG-014 and *UBA-1819*, and that all of them were negatively correlated with body weight and fat content, glucose, and inflammation markers [51]. The involvement of further mechanisms underlying the effects of resveratrol intake cannot be discarded. In this regard, resveratrol administration not only has been related to the modulation of gut microbiota composition, but also with improved intestinal integrity and production of microbial metabolites with well-known anti-inflammatory activity, including SCFAs [46]. In fact, gut-microbiota-derived metabolites elicit pleiotropic local effects in distinct organs, including the liver, through activation of diverse kinases [52]. Moreover, it has been reported that resveratrol tempers high-fat dietary induced steatohepatitis by preserving gut barrier disruptions and overt gut inflammation via the endocannabinoid system [53]. Similarly, the well-known anti-inflammatory effects of resveratrol [54] may also be involved in the hepatic benefits induced by the polyphenol administration, due to the relationship between microbiota dysbiosis and inflammation [55]. Therefore, all these processes could also explain the benefits observed after the administration of the polyphenol.

Regarding the strengths and limitations of this study, it must be highlighted that the selected experimental design and model is appropriate to elucidate the potential of resveratrol for diet-induced NAFLD prevention. Indeed, the diet used to generate NAFLD proved to be effective, as revealed by biochemical and histologic analysis, and should be considered a proof of principle. Nevertheless, this study also holds some limitations. Firstly, since this is a preclinical study, the potential translation of the observed results/effects to humans is not guaranteed. Additionally, faecal samples collected at the beginning of the study would enable better characterization of each animal according to baseline individual microbiota signature. Indeed, such information would be of interest to better establish the influence of the abundance of certain microbes in the response of the animals to resveratrol administration. Finally, these results, in terms of potential implication of specific bacteria in resveratrol mediated effects, derives from statistical associations. Since such are not causal inferences, and type I and II errors cannot be ruled out neither, these data should be completed and corroborated.

## 5. Conclusions

In conclusion, resveratrol administration effectively prevented high-fat high-fructose diet-induced NAFLD in a rodent model. In general, the abundance of bacteria from the *Ruminococcaceae* family seems to be negatively associated with hepatic markers including liver weight, serum transaminase levels and hepatic steatosis and inflammation degree. In the case of liver weight, the abundance of *UBA-1819* seems to have an interactive effect in resveratrol induced effects, which may differ depending on the faecal/gut occurrence of this bacteria.

Summing up, this investigation demonstrated a negative association among several gut microorganisms belonging to the *Ruminococcaceae* family with markers of liver status, while interestingly some markers showed a variable outcome depending on gut *Ruminococcaceae* content. Regarding resveratrol, *Ruminococcaceae* abundance elicit functional benefits of this polyphenol administration, which is of value when interpreting the putative interactive involvement of microbiota on resveratrol effects on NAFLD.

## Figures and Tables

**Table 1:** Liver steatosis degree and inflammation in rats fed a control diet (C), a high-fat high-fructose diet (HFHF) or a high-fat-high-fructose diet supplemented with 30 mg/kg/d resveratrol (RSV30). Values are presented as percentage.

Steatosis Degree (%)			
	**Control** (*n* = 10)	**HFHF** (*n* = 10)	**RSV30** (*n* = 10)
Grade 3	0	100	0
Grade 2	0	84	16
Grade 1	0	10	90
Grade 0	100	0	0
**Liver inflammation status (%)**			
	**Control** (*n* = 10)	**HFHF** (*n* = 10)	**RSV30** (*n* = 10)
Moderate	0	61	39
Mild	0	41	59
None	100	0	0
